# Sprains

**Published:** 1887-12

**Authors:** 


*Sprains.* By C. W. MANSELL MOULLIN, F.R.C.S. Pp. 268. London: H. K. LEWIS. 1887.

This is an exceedingly well written and useful monograph on an important and often neglected class of injuries. The author has evidently taken great pains in the preparation of his work, and has presented the whole subject in a form which is at once fresh, forcible, and scientific. There is a good deal of novelty in the work, both in observation and in reasoning; and much of sound sense in suggesting and criticising modes of treatment. One pleasant feature of the work is the style; clear, pithy, and elegant, evidently written by a man who has some acquaintance with and regard for the admitted usages of the English tongue.

